# Health-Related Quality of Life and Its Related Factors in Survivors of Stroke in Rural China: A Large-Scale Cross-Sectional Study

**DOI:** 10.3389/fpubh.2022.810185

**Published:** 2022-04-05

**Authors:** Yong-xia Mei, Zhen-xiang Zhang, Hui Wu, Jian Hou, Xiao-tian Liu, Sheng-xiang Sang, Zhen-xing Mao, Wei-hong Zhang, Dong-bin Yang, Chong-jian Wang

**Affiliations:** ^1^School of Nursing and Health, Zhengzhou University, Zhengzhou, China; ^2^Department of Epidemiology and Biostatistics, College of Public Health, Zhengzhou University, Zhengzhou, China; ^3^The People's Hospital of Hebi, Hebi, China; ^4^School of Public Health, Xinxiang Medical University, Xinxiang, China; ^5^School of Public Health, Tianjin Medical University, Tianjin, China; ^6^Henan Province General Medical Educations and Research Center, Xinxiang, China

**Keywords:** stroke, EQ-5D-5L, HRQoL, rural, influencing factors

## Abstract

**Background:**

Stroke is a major health threat and the leading cause of mortality and disability in China. The aims of this study were to identify the possible influencing factors of health-related quality of life (HRQoL) and its domain-specific contents in stroke patients in rural areas in China.

**Methods:**

A total of 1,709 stroke patients aged 36–79 years from the baseline data of Henan Rural Cohort study (*n* = 39,259) were included in the cross-sectional study. The Chinese version of the European Quality of Life Five Dimension (including mobility, self-care, usual activities, pain/discomfort, and anxiety/depression) Five Level Scale (EQ-5D-5L) and visual analog scale (VAS) were used to evaluate HRQoL in stroke patients. Tobit regression models, generalized linear models and binary logistic regression models were constructed to determine potential influencing factors of the EQ-5D utility index, as well as influencing factors of each domain and VAS score.

**Results:**

The mean utility index and VAS scores of stroke patients were 0.885 (SD, 0.204), and 68.39 (SD, 17.31), respectively. Pain/discomfort (PD, 35.2%) and mobility (MO, 30.4%) were the most frequently reported issues. Regression models revealed that illiterate; a low monthly income; low physical activity intensity; and diabetes, anxiety, depression, or poor sleep quality were significantly associated with lower utility index and VAS scores among stroke patients. In addition, patients with stroke who were older, female, drinking, smoking, and consuming a high-fat diet, had a higher BMI, and lived with a stroke for a longer time, were also significantly associated with different dimensions of the EQ-5D.

**Conclusion:**

Patients with stroke in rural areas in China had a low HRQoL. Factors associated with the EQ-5D utility index as well as each domain and VAS score, need to be considered by health providers in rural areas. Patients with stroke in rural areas need to be included in national basic public medical services and managed systematically by medical institutions.

## Introduction

Stroke is a major health threat and the leading cause of mortality and disability in the world, with substantial economic costs associated with post-stroke care (1). Those living in East Asia have the highest risk of stroke incidence in the world, with a risk of 38.8% (2). China has the highest risk of stroke incidence among all countries, with a 39.3% risk, and Chinese men have the highest risk of stroke incidence of any male population worldwide, with a more than 41% risk (2). China accounted for 5.51 million (40%) of the 13.7 million strokes worldwide in 2016 (3). The mortality-to-incidence ratio of stroke has regional differences (4). In addition, approximately 90% of survivors with stroke have compromised functions (5), causing physical, psychological, and social disabilities and adding to the financial burden on survivors and their families (6, 7). Moreover, the burden of stroke appears to be increasing disproportionately in rural areas because the stroke incidence in rural areas (298 cases per 100,000 person-years) is higher than that in urban areas (204 cases per 100,000 person-years) in China (8).

Health-related quality of life (HRQoL) has been clearly shown to affect health, and includes perceptions of both physical and mental health and their correlations on the individual level, according to the National Center for Chronic Disease Prevention and Health Promotion (9). Living with the effects of stroke can influence a patient's physical and psychological health, leading to decreased HRQoL (10). The HRQoL of persons living with the effects of stroke was reported to be lower than that of people living without the effects of stroke (11). It is becoming more important to monitor and improve HRQoL in patients with stroke due to the improved survival following successful acute management (4).

The contextual model of HRQoL comprises macro and micro levels. The macro level, includes demographic factors, such as age and gender; the micro level, includes the individual's general health condition and comorbidities, health knowledge, motivation to engage in health-related behavior; and psychological factors such as the presence of anxiety, depression (12, 13). Identification of influencing factors related to HRQoL can guide effective strategies for HRQoL improvement in patients with stroke. Previous studies that focused on HRQoL showed that the predictors of HRQoL included demographic factors, clinically related factors, and environmental and individual factors; however, the results have been inconsistent due to cultural differences (14). Moreover, the use of different tools for measuring HRQoL may also lead to the identification of different factors associated with HRQoL (15). The European Quality of Life Five Dimensions (EQ-5D), composed of five different health dimensions (mobility, self-care, usual activities, pain/discomfort, and anxiety/depression), is a measure closely related to HRQoL that includes a broad multidimensional concept (16). The instrument is one of the simplest and most commonly used self-reporting instruments to evaluate HRQoL due to its simple, low response requirements, and generally high acceptance in population health surveys (17); the EQ-5D 5 Level (EQ-5D-5L) can reduce the ceiling effect more than the EQ-5D-3L, and has some psychometric advantages over the EQ-5D-3L in measuring some generic health outcomes in patients with stroke (18–20). However, most researchers have focused on only the influencing factors of the total EQ-5D score, and have paid little attention to the influencing factors of each domain score (21, 22). Due to the multidimensionality of HRQoL, understanding the influencing factors of the scores of each domain of the EQ-5D-5L will provide detailed insight into the challenges of survivors of stroke (21).

Henan is a populous province located in central China, with half of the total population living in rural areas (23). People living in rural areas generally have worse HRQoL than those living in urban areas (24). Data on the HRQoL and specific domains in patients with stroke in rural Henan remain limited. To guide effective and useful interventions to improve HRQoL in rural patients with stroke, this study aimed to evaluate HRQoL, its specific domains, and possible determinants in a large sample of rural patients with stroke using the Chinese EQ-5D-5L.

## Materials and Methods

### Settings and Participants

The study population was from the Henan Rural Cohort study (25), which was conducted in five rural regions from different geographical regions Suiping County, Yuzhou County, Xinxiang County, Tongxu County, and Yima County, which are located in South, North, East, West, and Middle Henan Province, China, and the baseline survey was carried out between July 2015 and September 2017. A multistage, stratified cluster sampling method was used to obtain samples in the general population. The target population was adults aged 18–79 years who were permanent residents and available to complete follow-up mortality and morbidity studies.

The respondents were requested to complete a standard questionnaire including information on general demographic characteristics, lifestyle, physical activity, sleep quality, mental health and health state. They were also asked to report their health status using the EQ-5D-5L. A detailed description of the survey in the Henan rural cohort has been published elsewhere (25).

A total of 39,259 people responded to the survey and 6.7% of them (2,642) reported suffering from stroke. The inclusion criteria of stroke survivors were self-reported medical histories of stroke, and confirmation by village doctors according to their New Rural Cooperative Medical System medical records (each participant had a unique medical insurance card number and ID, making it easy to track disease). Further identification of medical histories was performed by an outcome committee consisting of an internist, an endocrinologist, a cardiologist, and an epidemiologist according to standards recommended by the World Health Organization (26). The exclusion criteria were stroke survivors who did not complete EQ-5D-5L information (the details can be found online in Supplementary Table 1); thus, the final dataset in this study contained data for 1,709 survivors of stroke.

### Patient and Public Involvement

Neither patients with stroke nor the public were involved in the study design or contributed to the writing or editing of this document for readability or accuracy.

### Ethics Consideration

This study was approved by the Zhengzhou University Life Science Ethics Committee under ethical approval code: [2015] MEC (S128), and written informed consent was obtained from all participants.

### EQ-5D-5L

The Chinese version of the EQ-5D-5L, a generic preference-based instrument, consists of a descriptive system and EQ-5D visual analog scale (EQ-VAS) (27). The descriptive system comprises five domains (mobility, self-care, usual activities, pain/discomfort, and anxiety/depression), and each dimension has five levels of severity (no problems, slight problems, moderate problems, severe problems and extreme problems) (16). The responses for the five dimensions can be combined in a 5-digit number describing the respondent's health state (from 11,111 meaning no problems at all to 55,555 meaning extreme problems in all five dimensions, a total of 3,125 possible health states are were defined in this way) (28). An EQ-5D utility index is derived by applying a formula that attaches values (weights) to each of the levels in each dimension. The index is calculated by deducting the appropriate weights from 1, the value representing full health which are represented by a single “utility” index ranging from −0.391 (for 55,555) to 1.000 for (11,111). The responses for the five dimensions are represented by a single “utility” index ranging from −0.391 (for 55,555) to 1.000 (for 11,111) considering Chinese crosswalk value sets (29). A score index of 1 represents full health, a score of 0 represents death, and a score <0 represents a health status worse than death (29). The EQ-VAS asks people to rate their health state on a vertical thermometer-like scale ranging from best imaginable health (100) to worst imaginable health (0) (16). The intraclass correlation coefficients ranged from 0.69 to 0.93 for the EQ-5D-5L, which indicated moderately better distributional parameters and substantial improvement in informativity compared that of the 3L (28).

### Participants' Characteristics

The sociodemographic variables (e.g., age, sex, marital status, education, and income), lifestyle variables (e.g., smoking and alcohol consumption statuses), and duration of illness (years, how long since the first stroke) are shown in Table 1 for patients with stroke. Moreover, consumption of fats, vegetables and fruit were collected with a food frequency questionnaire in person which food items were converted to grams per day for analysis in accordance with the dietary guidelines for Chinese residents (30). A high-fat diet was defined as the diet of a person who consumed an average of more than 75 g of livestock and poultry meat per day; a high vegetable and fruit diet was defined as the diet of a person consuming at least 500 g of vegetables or fruits per day. Physical activity intensity information over the last 7 days was collected with the International Physical Activity Questionnaires (short form) (test-retest reliability was ~0.8, and correlation coefficients were above 0.65) and categorized into three levels (light, moderate, and vigorous) (31, 32).

**Table 1:** Definitions and coding schema for patients with stroke' sociodemographic variables.

**Variables**	**Definitions and coding schema**
Age	<55 = 1; 55~= 2; ≥65 = 3
Sex	Male = 1; Female = 2
Marital status	Categorized as married/cohabiting and widowed/unmarried/divorced/separated married/cohabiting = 1; widowed/unmarried/divorced/separated = 2
Spouse	Defined as whether a patient with stroke was accompanied by a spouse and categorized as yes or no No = 0; Yes = 1
Educational level	Categorized as illiterate, primary school, junior high school, or above Illiterate = 1; Primary school = 2; Junior high school and above = 3
Per capita monthly actual income	Categorized as < $72 (US dollars), $72 ~ $143, and >$143 <72 = 1; 72~= 2; ≥143 = ~3
Smoking status	Defined as at least one cigarette per day for the previous 6 months Never = 0; Current = 1; Former = 2
Drinking status	Defined as alcohol consumption 12 times per year Never = 0; Current = 1; Former = 2
Duration of illness (years)	Defined as how long since the first stroke and categorized as <1, ≥1 to <3, ≥3 to <5, or ≥5 years <1 year = 1; ≥1 to <3 years = 2, ≥3 to <5 years = 3, or ≥5 years = 4

Body mass index (BMI) was calculated as weight (kilogram) divided by height (meters squared), and was divided into four categories: underweight (<18.5 kg/m^2^), normal weight (18.5 kg/m^2^ ≤ BMI < 24.0 kg/m^2^), overweight (24.0 kg/m^2^ ≤ BMI < 28.0 kg/m^2^) and obese (BMI ≥ 28.0 kg/m^2^) (31). Centripetal obesity was diagnosed when the waist circumference (WC) was ≥90 cm in males and ≥80 cm in females (33). A normal waist-to-hip ratio was defined as a ratio ≥0.9 in males and ≥0.8 in females (34). The normal waist-to-height ratio was defined as ratio ≥0.50 (35). Hypertension was defined as blood pressure ≥140/90 mmHg or the use of antihypertensive medication (36). Diabetes mellitus was defined as fasting blood glucose ≥7.0 mmol/L, a self-reported diagnosis by a doctor, or the use of hypoglycemic agents including insulin. Anthropometric variables were measured while the participants wore light clothing, and body weight and height to the nearest 0.1 kg and 0.1 cm, respectively, were measured twice. Blood pressure was measured using an electronic sphygmomanometer (HEM-770A Fuzzy, Omron, Japan) while the patient was in a seated position after 5 min of rest, and fasting blood glucose was measured by analyzing venous blood samples after overnight fasting.

Anxiety in the previous 2 weeks was measured by the 2-item Generalized Anxiety Disorder Scale (GAD-2), with a GAD-2 score ≥3 indicating anxiety (with a sensitivity of 76% and specificity of 81% for major anxiety), which has been validated in multiple studies and shown to have excellent psychometric properties (37). Depression in the previous 2 weeks was measured by the 2-item Patient Health Questionnaire-2 (PHQ-2), which is a brief depression screening measure and has good validity, with a PHQ-2 score ≥2 indicating depression (with a sensitivity of 83% and a specificity of 92% for major depression) (38). Sleep quality in the previous month was measured by the Pittsburgh Sleep Quality Index (PSQI) (with a test–retest reliability of 0.85), with a PSQI score >5 indicating poor sleep quality (the sensitivity was 98% using a cutoff value of 5 to discriminate poor sleepers from good sleepers) (39). More detailed information on the cohort has been described elsewhere (25).

### Data Analysis

Data were analyzed using STATA 15 for Windows and the IBM SPSS statistics software package, version 21.0 for windows, for Windows (Chicago, IL, USA). Means and standard deviations (SDs) are used to describe continuous variable data, and frequencies and percentages are used to describe categorical variable data. The associations between the EQ-5D domains and the various participants' characteristics were tested by chi-square tests in the univariate analysis. Binary logistic regressions was used to assess the potential influencing factors of EQ-5D domain scores (which were classified as no problem and having problems). Mann–Whitney *U* (two groups) and Kruskal–Wallis one-way analysis of variance (multiple groups) were used to examine the associations between all the various participant characteristics and the EQ-5D-5L utility index and VAS scores due to the abnormal distribution of the EQ-5D utility index and VAS score. A Tobit regression model was constructed to explore the potential influencing factors of the EQ-5D-5L utility index score. These methods were chosen because the EQ-5D utility data were skewed and the utility score was censored at 1 (40). A generalized linear model (GLM) was applied to determine the potential influencing factors of EQ-5D-5LVAS scores. These methods were chosen because the VAS score data were not normally distributed. Statistical significance was set at *P* ≤ 0.05 in all analyses.

## Results

### Sample Characteristics

The mean (SD) age of the sample was 64.06 years (7.63), with ages ranging from 36 to 79 years. The characteristics of the patients with stroke are shown in Table 2.

**Table 2:** Distribution of stroke patients within different subgroups over reporting problems (slight to extreme problems) of the EQ-5D dimension, mean (SD) EQ−5D utility index, and VAS score (*n* = 1,709).

**Subject**	***n* (%)**	**Mobility**	**Self-care**	**Usual**	**Pain/**	**Anxiety/**	**Mean utility**	**Mean VAS**
**characteristics**				**activities**	**discomfort**	**depression**	**index (SD)**	**scores (SD)**
**Age(years)**								
<55	237 (13.90)	46 (19.40)^**^	21 (8.90)^**^	29 (12.20)^**^	67 (28.30)^*^	24 (10.10)	0.92 (0.16)^**^	69.58 (17.84)
55 ~ 65	580 (33.90)	155 (26.70)	54 (9.30)	88 (15.20)	191 (32.90)	58 (10.00)	0.90 (0.18)	68.97 (17.58)
65~	892 (52.20)	319 (35.80)	147 (16.50)	215 (24.10)	343 (38.50)	120 (13.50)	0.86 (0.23)	67.70 (16.98)
*P*		<0.001	<0.001	<0.001	0.005	0.092	<0.001	0.203
**Gender**								
Female	905 (53.00)	266 (29.40)	116 (12.80)	175 (19.30)	356 (39.30)^**^	133 (14.70)^**^	0.87 (0.21)^*^	68.17 (17.76)
*P*		0.324	0.822	0.921	<0.001	<0.001	0.005	0.583
**Education**								
Illiterate	425 (24.90)	149 (35.10)^*^	66 (15.50)	97 (22.80)^*^	175 (41.20)	60 (14.10)^*^	0.86 (0.22)^**^	67.72 (18.45)^*^
Primary school	596 (34.90)	194 (32.60)	82 (13.80)	129 (21.60)	232 (38.90)	77 (12.90)	0.87 (0.22)	67.18 (17.15)
Other	688 (40.30)	177 (25.70)	74 (10.80)	106 (15.40)	194 (28.20)	65 (9.40)	0.91 (0.18)	69.85 (16.61)
*P*		0.002	0.056	0.002	0.100	0.038	<0.001	0.014
**Spouse**								
Yes	1,458 (85.30)	436 (29.90)	191 (13.10)	279 (19.10)	508 (34.80)	168 (11.50)	0.88 (0.21)	68.43 (17.17)
*P*		0.257	0.744	0.464	0.498	0.359	0.812	0.688
**Per capita monthly actual income ($)**								
<72	801 (46.90)	282 (35.20)^**^	126 (15.70)^*^	187 (23.30)^**^	316 (39.50)^*^	112 (14.00)^*^	0.86 (0.22)^**^	65.79 (17.63)^**^
72 ~ 143	500 (29.30)	150 (30.00)	61 (12.20)	96 (19.20)	158 (31.60)	53 (10.60)	0.90 (0.19)	69.63 (16.61)
143~	408 (23.90)	88 (21.60)	35 (8.60)	49 (12.00)	127 (31.10)	37 (9.10)	0.92 (0.18)	71.97 (16.76)
*P*		<0.001	0.012	<0.001	0.013	0.020	<0.001	<0.001
**Smoking status**								
Never	1,140 (66.70)	346 (30.40)	157 (13.80)	233 (20.40)	433 (38.00)^*^	166 (14.60)^**^	0.87 (0.21)^*^	67.92 (17.54)
Former	276 (16.10)	91 (33.00)	36 (13.00)	51 (18.50)	82 (29.70)	15 (5.40)	0.91 (0.18)	70.00 (16.81)
Current	293 (17.10)	83 (28.30)	29 (9.90)	48 (16.40)	86 (29.40)	21 (7.20)	0.89 (0.18)	68.63 (16.83)
*P*		0.483	0.213	0.267	0.003	<0.001	0.007	0.179
**Drink status**								
Never	1,287 (75.30)	384 (29.80)^*^	172 (13.40)^*^	253 (19.70)^*^	464 (36.10)	174 (13.50)^*^	0.88 (0.21)^*^	67.96 (17.19)^**^
Former	205 (12.00)	87 (40.10)	37 (17.10)	55 (25.30)	76 (35.00)	14 (6.50)	0.92 (0.17)	73.70 (15.57)
Current	217 (12.70)	49 (23.90)	13 (6.30)	24 (11.70)	61 (29.80)	14 (6.80)	0.88 (0.18)	66.26 (18.82)
*P*		0.001	0.003	0.002	0.215	0.001	0.024	<0.001
**High-fat diet**								
Yes	166 (9.70)	40 (24.10)	13 (7.80)^*^	21 (12.70)^*^	56 (33.70)	18 (10.80)	0.91 (0.16)	70.93 (16.18)^*^
*P*		0.062	0.037	0.020	0.684	0.682	0.079	0.046
**Vegetable and fruit diet**								
Yes	722 (42.20)	184 (25.50)^**^	86 (11.90)	119 (16.50)^*^	236 (32.70)	65 (9.00)^*^	0.90 (0.19)^*^	69.59 (16.90)^*^
*P*		<0.001	0.257	0.009	0.066	0.002	0.007	0.014
**Physical activity intensity**								
Mild	748 (43.80)	306 (40.90)^**^	154 (20.60)^**^	213 (28.50)^**^	285 (38.10)	97 (13.00)	0.84 (0.25)^**^	67.32 (17.68)
Moderate	536 (31.40)	131 (24.40)	51 (9.50)	80 (14.90)	180 (33.60)	63 (11.80)	0.91 (0.16)	68.39 (18.11)
Intense	425 (24.90)	83 (19.50)	17 (4.00)	39 (9.20)	136 (32.00)	42 (9.90)	0.93 (0.11)	70.26 (15.41)
*P*		<0.001	<0.001	<0.001	0.071	0.290	<0.001	0.201
**BMI (*****n*** **=** **1,244)**								
<18.5	37 (2.19)	13 (35.10)	4 (10.80)	6 (16.20)	14 (37.80)	11 (29.70)^*^	0.85 (0.26)	62.14 (21.19)
18.5 ≤ BMI <24.0	587 (34.67)	164 (27.90)	72 (12.30)	113 (19.30)	210 (35.80)	63 (10.70)	0.89 (0.19)	67.98 (16.60)
24.0 ≤ BMI <28.0	739 (43.65)	212 (28.70)	84 (11.40)	127 (17.20)	244 (33.00)	86 (11.60)	0.90 (0.19)	69.02 (17.30)
≥28.0	330 (19.49)	118 (35.80)	51 (15.50)	74 (22.40)	126 (38.20)	38 (11.50)	0.87 (0.22)	68.86 (17.86)
*P*		0.058	0.304	0.230	0.388	0.007	0.121	0.852
**Centripetal obesity (*****n*** **=** **1,245)**								
Yes	917 (54.00)	293 (32.00)	119 (13.00)	182 (19.80)	322 (35.10)	114 (12.40)	0.88 (0.20)	68.79 (17.20)
*P*		0.073	0.622	0.391	0.995	0.328	0.160	0.389
**Waist-to-hip ratio (*****n*** **=** **1,244)**								
abnormal	1,268 (74.72)	124 (28.90)^*^	49 (12.20)^**^	74 (18.50)	141 (35.20)	41 (10.20)	0.88 (0.20)	68.64 (17.10)
*P*		0.021	<0.001	0.705	0.970	0.283	0.385	0.451
**Duration of the illness (years)**								
<1	209 (12.20)	54 (25.80)^**^	25 (12.00)^*^	32 (15.30)^*^	76 (36.40)	25 (12.00)	0.90 (0.17)	70.91 (16.38)
≥1– <3	486 (28.40)	123 (25.30)	52 (10.70)	76 (15.60)	161 (33.10)	52 (10.70)	0.90 (0.19)	69.09 (17.35)
≥3– <5	347 (20.30)	97 (28.00)	36 (10.40)	63 (18.20)	121 (34.90)	38 (11.00)	0.90 (0.19)	68.39 (17.97)
≥5	663 (38.8)	245 (37.00)	109 (16.40)	161 (24.30)	241 (36.30)	86 (13.00)	0.86 (0.23)	67.12 (17.18)
*P*		<0.001	0.009	0.001	0.697	0.640	0.200	0.320
**Hypertension**								
Yes	727 (42.50)	258 (35.50)^**^	108 (14.90)^*^	163 (22.40)^*^	248 (34.10)	84 (11.60)	0.87 (0.21)^*^	66.93 (16.80)^*^
*P*		<0.001	0.048	0.007	0.432	0.770	0.049	0.003
**Diabetes mellitus**								
Yes	236 (13.80)	95 (40.30)^**^	39 (16.50)	60 (25.40)^*^	85 (36.00)	32 (13.60)	0.84 (0.27)^**^	66.58 (16.97)
*P*		<0.001	0.082	0.012	0.768	0.373	<0.001	0.083
**Anxiety (*****n*** **=** **1246)**								
GAD-2 ≥ 3	154 (9.00)	71 (46.10)^**^	40 (26.00)^**^	54 (35.10)^**^	108 (70.10)^**^	76 (49.40)^**^	0.71 (0.32)^**^	60.39 (19.09)^**^
*P*		<0.001	<0.001	<0.001	<0.001	<0.001	<0.001	<0.001
**Depression (*****n*** **=** **1,246)**								
PHQ-2	183 (10.70)	96 (52.50)^**^	51 (27.90)^**^	76 (41.50)^**^	121 (66.10)^**^	87 (47.50)^**^	0.69 (0.33)^**^	59.75 (20.07)^**^
*P*		<0.001	<0.001	<0.001	<0.001	<0.001	<0.001	<0.001
**Sleep quality (*****n*** **=** **1,229)**								
PSQI > 5	513 (30.02)	320 (26.40)^**^	120 (9.90)^**^	188 (15.50)^**^	353 (29.10)^**^	100 (8.30)^**^	63.78 (17.31)^**^	0.82 (0.26)^**^
*P*		<0.001	<0.001	<0.001	<0.001	<0.001	<0.001	<0.001

*GAD-2, Generalized anxiety disorder scale-2; PHQ-2, Patient health questionnaire-2; PSQI, the Pittsburgh sleep quality index;*

* *P < 0.05*;

** *P < 0.001*.

### Reported Health Problems

Regarding the EQ-5D-5L, pain/discomfort was the most frequently reported issue (35.2%), followed by mobility (30.4%). anxiety/depression was the least reported issue (11.8%). The frequencies of pain/discomfort, mobility, usual activities, anxiety/depression, and self-care were all significantly different among the monthly income groups and among those with anxiety, depression, and poor sleep quality (Table 2). In addition, the frequencies of the different issues were significantly different among survivors of stroke with different conditions, as shown in Table 2.

### EQ-5D-5L Utility Index and VAS Scores

**Figure 1:**
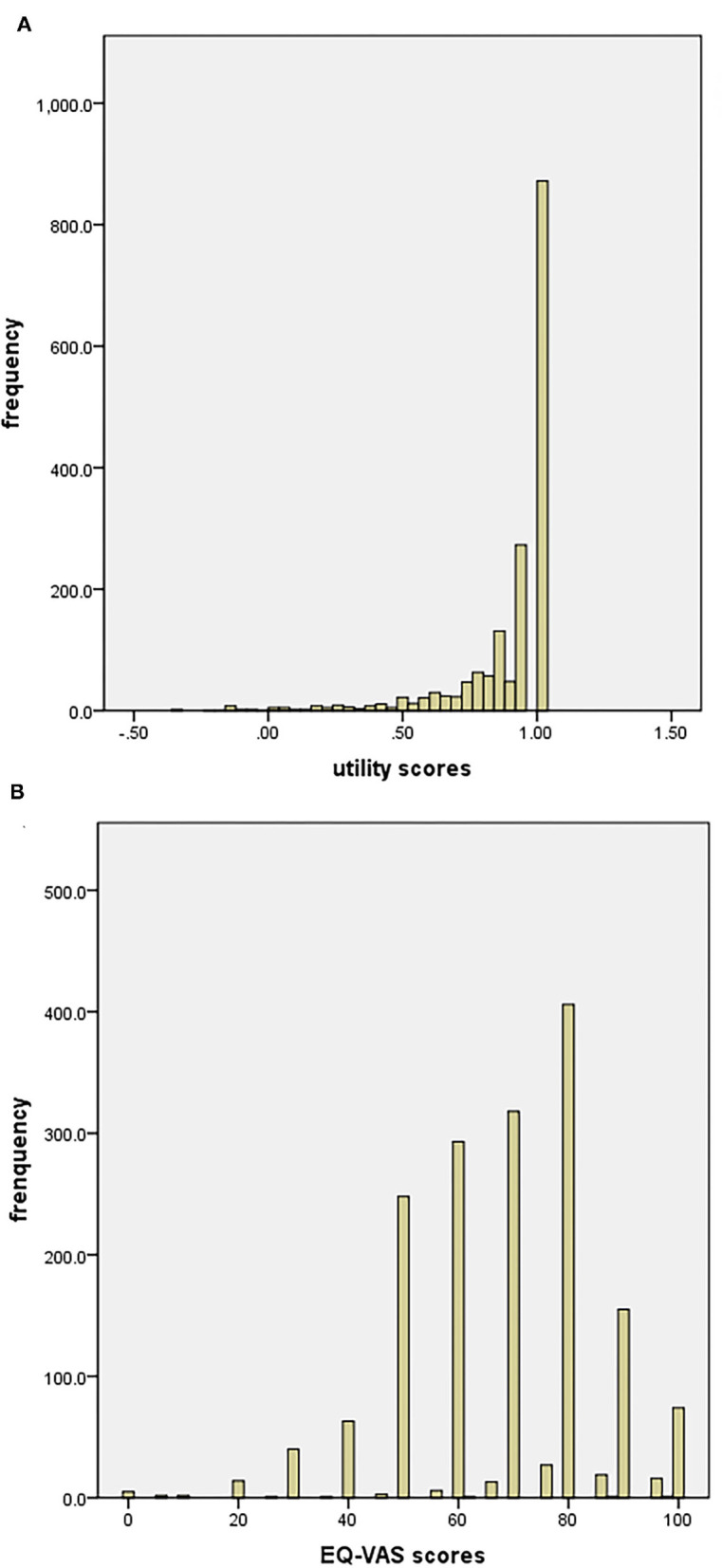
**(A)** Distribution of EQ-5D-5l scores (*n* = 1,709); **(B)** distribution of EQ-VAS scores (*n* = 1,709).

The mean utility index of patients with stroke was 0.885 (SD, 0.204), ranging from −0.348 to 1.000 with a left-skewed distribution (skewness = −2.769) (Figure 1A). The mean VAS score of the participants was 68.39 (SD, 17.31), with skewness of −0.533 (Figure 1B).

The EQ-5D-5L utility index and VAS scores stratified by different participant characteristics are presented in Table 2. The differences in the EQ-5D-5L utility index and VAS scores among the education level groups, monthly income groups, alcohol consumption groups, vegetable and fruit diet groups, hypertension and anxiety groups, depression groups, and poor sleep quality groups were statistically significant (*P* < 0.05; Table 2).

### Factors Associated With Each EQ-5D Dimension Score

**Figure 2:**
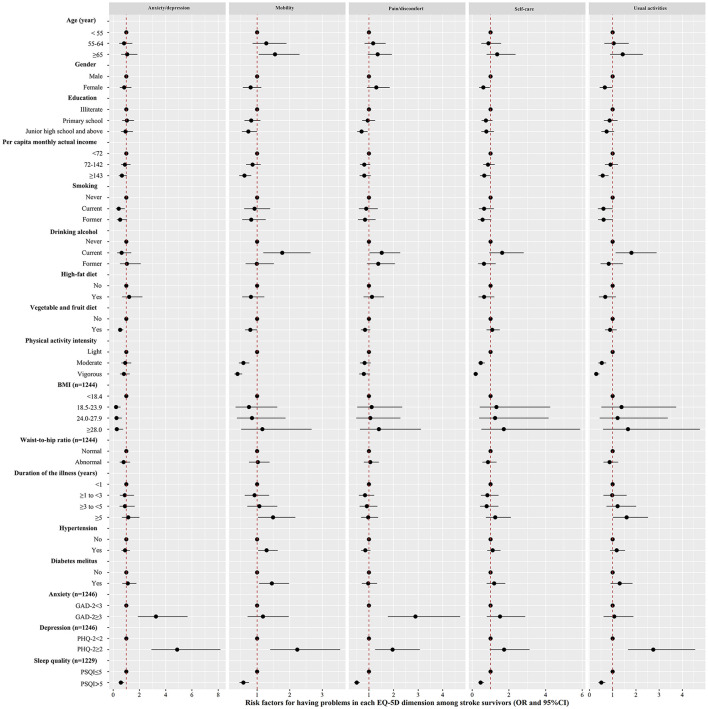
The risk factors for low scores in each EQ-5D dimension [Mobility, Self-care (SC), Usual activities (UA), Pain/discomfort and Anxiety/depression (AD)] among patients with stroke (*n* = 1,709) identified by multiple logistic regression analysis. The black dots with the corresponding error bars represented the estimated effect and 95% confidence intervals of the change in each of variables. A two-sided *P*-value 0.05 was considered statistically significant.

The risk factors for low scores in each EQ-5D dimension identified by multiple logistic regression analysis are shown in Figure 2, and the details can be found online in Supplementary Table 2. Older patients with stroke reported more mobility issues than younger patients. Female survivors of stroke were less likely to participate in self-care and usual activities than male survivors of stroke. Patients with stroke with a higher education level were less likely to have mobility issues and pain/discomfort issue than those with a lower education level. Patients with stroke with higher income were less likely to have mobility issues and more likely to perform usual activities than those with a lower income.

Patients with stroke who smoked reported less anxiety/depression than those who never smoked. Patients with stroke who drank alcohol reported more mobility issues, more usual activities and more pain/discomfort than those who never drank alcohol. Patients with stroke who consumed a high-fat diet were less likely to have anxiety/depression issues than those who did not consume a high-fat diet. The higher the BMI was the fewer anxiety/depression problems patients reported. Patients with stroke who reported intense physical activity had fewer mobility issues, performed less self-care and performed less usual activities than those with less intense physical activity. Patients who had lived with the effects of stroke for more than 5 years reported more mobility issues and performed more usual activities than those who had lived with the effects of stroke for <1 year.

Patients with stroke with anxiety were more likely to have issues with pain/discomfort and anxiety/depression than those without anxiety. Patients with stroke with depression had a higher risk of mobility issues, usual activity issues, pain/discomfort, and anxiety/depression issues than those without depression. Patients with stroke with good sleep quality reported fewer issues with mobility, self-care, usual activities, pain/discomfort, and anxiety/depression than those with poor sleep quality.

### Factors Associated With the EQ-5D-5L Utility Index and VAS Scores

**Figure 3:**
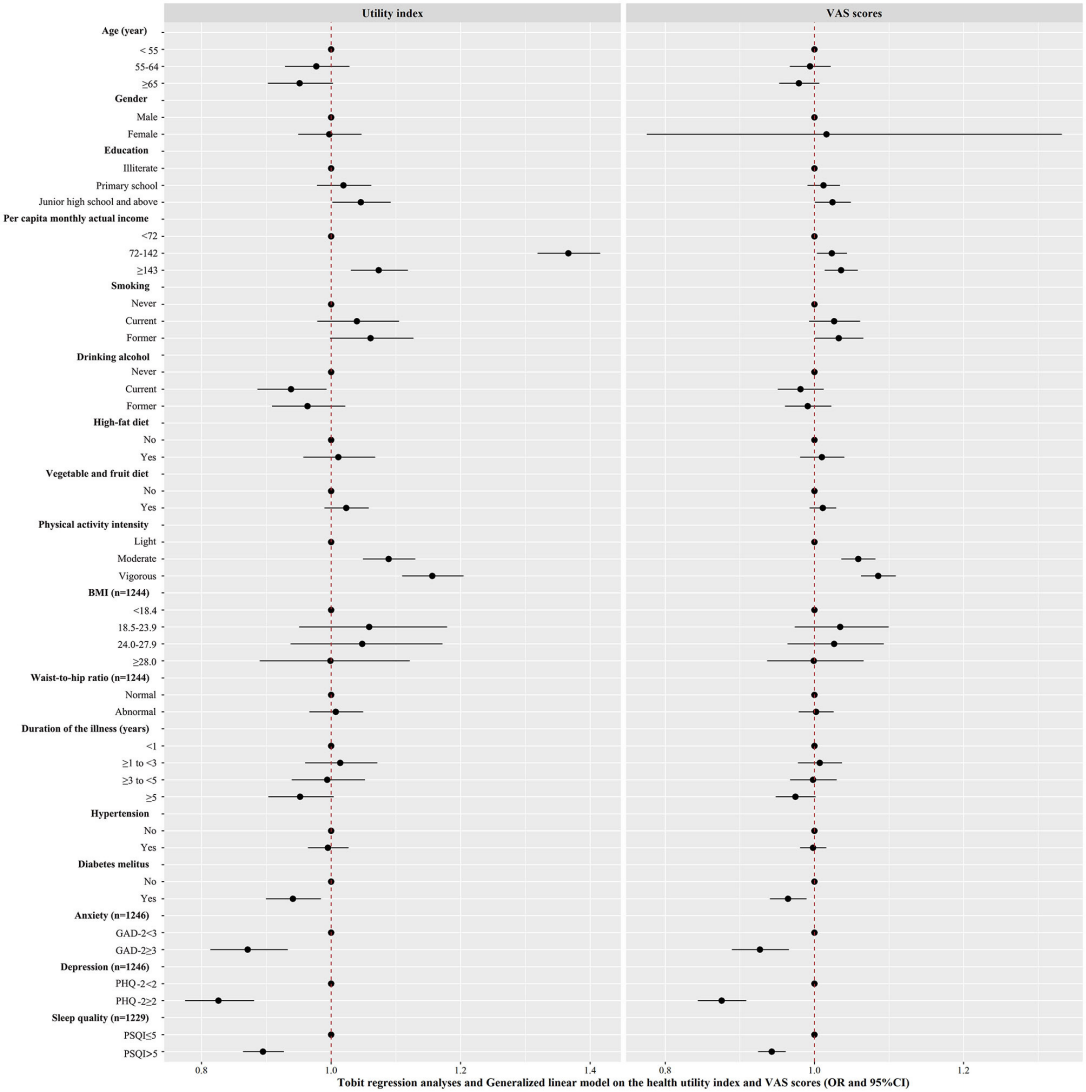
Factors associated with EQ-5D-5L utility index and Visual Analogue Scale scores in patients with stroke were analyzed by Tobit regression and Generalized linear model, respectively (*n* = 1,709). The estimated effect and 95% confidence intervals were represented by black squares with the corresponding error bars. A two-sided *P*-value 0.05 was considered statistically significant.

The influencing factors of the health utility index and VAS scores according to the Tobit regression analyses and GLM are shown in Figure 3, and the details can be found online in Supplementary Table 3. Patients with stroke who were smokers had higher VAS scores (but not utility index scores) than nonsmokers. Patients with stroke who consumed alcohol had a lower utility index (but not VAS scores) than those who did not. Patients with stroke who were illiterate; had a lower monthly income; reported lower physical activity intensity; and had diabetes, anxiety, depression, and poor sleep quality had significantly lower health utility index and VAS scores.

## Discussion

Our study enriches the current literature on the relationship among the EQ-5D utility index, the domain-specific scores, and the factors associated with each in a large sample of patients with stroke in rural areas. The main findings were that the most frequently reported problems were in the pain/discomfort dimension, and patients with stroke who were illiterate, had a lower monthly income, reported lower physical activity intensity, and had diabetes, anxiety, depression and poor sleep quality were significantly associated with lower health utility index and VAS scores. In addition, patients with stroke who were older, female, drinking, smoking, consuming a high-fat diet, having a higher BMI, and living with stroke for a longer time were also significantly associated with different dimensions of the EQ-5D.

The mean utility index (0.885) and VAS (68.39) scores in patients with stroke were much lower than those (EQ index of 0.957 and VAS score of 86.0) in the urban Chinese population (41), and were also lower than those in a rural population with dyslipidemia (EQ index as 0.953 and VAS score as 78.46) in Xinxiang, Henan (42). This result indicated that stroke had a great impact on decreased HRQoL in a Chinese rural population (43). However, the mean utility index and VAS scores were higher than those in patients with stroke in Canada (EQ index of 0.79) (44). The reason for this may be that the patients in Canada were undergoing inpatient rehabilitation, typically commencing 1–4 weeks post-stroke. The mean utility index in our study was higher than that in patients with stroke in Korea (EQ index of 0.757) (11), and the mean VAS (68.39) scores were higher than those in patients with stroke in Europe (VAS score as of 63.74) (45). The reason for this may be the different crosswalk value sets used as well as cultural and social distinctions (14, 46). This finding suggests that stroke patients from different cultures should be targeted.

Our findings showed that the most frequently reported problems of patients with stroke were in the pain/discomfort dimension. Pain after stroke is a common clinical problem that not only increases depression and cognitive issues but also impairs quality of life (47). Moreover, pain after stroke is usually underdiagnosed and undertreated (47). Health care workers need to assess the pain early and help patients obtain relief. Our results indicated the importance of pain problems in patients with stroke living in urban areas. This finding was consistent with people with dyslipidemia in Henan (pain/discomfort was the most frequently reported issue, at 20.83%) (42), but did not agree with the results of a study in Taiwan, in which the most frequent issue of patients with stroke was self-care, and 30% of patients with stroke reported pain/discomfort issues (48). A possible reason for this may be the higher number of multiple chronic diseases in rural areas and the aging population of rural residents (49) or the difference in study design. This finding is inconsistent with the results of a study in Australia, in which nearly 50% of reported problems (43% moderate; 7% extreme) are in the EQ-5D-3L anxiety or depression domain (50).This may be because cultural diversity or the measurements are different, and the EQ-5D-5L used in this study reduced ceiling effects compared with the EQ-5D-3L (28).

Patients with stroke with higher education levels (associated with fewer mobility and pain/discomfort issues) and monthly income (associated with fewer mobility and usual activities issues) had higher health utility index and VAS scores than those with lower education levels and monthly incomes; education and income are known as predictors of quality of life in patients with stroke (10, 11). This highlights the importance of ensuring equity in health opportunities for patients with stroke. The Chinese government is committed to implementing “well-off society programs” to help improve the incomes, education levels and quality of life of the population (51). Although the program has not yet translated to increased HRQoL in patients with stroke, the situation will improve with improved economic and educational statuses in the future.

Physical activity plays an important role in the prevention of stroke recurrence and improvement in quality of life (52). Patients with stroke who reported lower physical activity intensity had lower health utility index and VAS scores than those who reported high physical activity intensity, and the less intense physical activity they performed, the more mobility, self-care, and usual activities issues they reported. Although the degree of motor impairment was not considered in this study, the level of physical activity among patients with stroke in rural areas still needs to be improved. Evidence supports the fact that survivors of stroke who perform various kinds of training, such as aerobic, strength, and traditional Chinese exercises, have an improved quality of life (53). However, the different intensities and amounts of physical activity for heterogeneous stroke populations need to be explored further.

Patients with stroke with diabetes had lower health utility index and VAS scores and were more likely to have pain/discomfort and anxiety/depression issues than those without diabetes. Patients suffering from comorbidities (such as diabetes) had a significantly worse EQ-5D profile (54). Cardiovascular disease comorbid with diabetes has direct effects on quality of life, health service demands and economic costs (55). This finding indicates that more attention should be given to stroke patients with diabetes.

Patients with stroke with anxiety and depression had lower health utility index and VAS scores. Patients with higher level of depression, anxiety and disability were associated with lower HROoL scores after stroke at 5 years (45). Anxiety has both direct and indirect effects on quality of life in patients with stroke (56). Anxiety is common during the first year after stroke; it significantly influences quality of life and is a predictor of depression (57). Depression significantly decreased the EQ-5D utility index and VAS scores. Post-stroke depression is one of the most common complications that negatively affects quality of life (58). Post-stroke depression is associated with worse recovery and outcomes in multiple functional domains such as activity limitations and cognitive deficits, which leads to worse HRQoL in patients with stroke (59). In addition, previous research has shown that elderly individuals in rural areas exhibit more depression symptoms than those in urban areas (60). Therefore, routine anxiety and depression screening and non-pharmacological treatments, such as life review therapy, problem-solving therapy, and exercise, should be provided in rural stroke rehabilitation programs (61). Moreover, a study showed that women living in rural areas had a higher prevalence of depression disorders than those living in urban areas (62); thus, additional attention needs to be paid to female patients with stroke and interventions should be sex-specific.

A study showed that adults with stroke had poorer sleep quality than age-and sex-matched adults without stroke (63). Poor sleep quality in patients with stroke should be considered when developing a rehabilitation plan. Moreover, sleep time is one of the key factors affecting quality of life improvement in patients with stroke; 6–8 h of sleep each day is recommended (64). However, evidence-based sleep recommendations, such as how to achieve 6–8 h of sleep per day, are needed from our health organizations. In addition, physical activity can affect the relationship between sleep quality and quality of life in patients with stroke (65), and further research needs to be performed on how physical activity affects this relationship.

Older patients with stroke reported a higher rate of mobility issues than younger patients with stroke. Additionally, patients with stroke who smoke or drink take for granted that they have a higher HRQoL due to smoking or drinking. They do not realize the harm of smoking or drinking, so health education about a healthy lifestyle should be enhanced (26).

### Implications for Clinical and Research Practice

This study reported the influencing factors of the EQ-5D utility index and its domain-specific scores in patients with stroke based on a representative rural population in China, which guaranteed the reliability. The findings provide important implications. First, this study highlights the importance of the HRQoL of patients with stroke in rural areas, which should encourage the government to focus more on the systematic management of patients with stroke in rural areas. Patients with stroke with a low level of HRQoL need more assistance and resources. Second, the influencing factors of the EQ-5D utility index and the domain-specific scores in patients with stroke provide a more detailed insight into potential interventions to improve the HRQoL of patients with stroke. Interventions focusing on maintaining healthy lifestyles and improving mental health and sleep quality may be designed by nurses, according to diverse patients with stroke populations and various settings, to improve HRQoL.

### Limitations

However, several limitations in this study should be noted. First, the stroke survivors were from the Henan Rural Cohort study, which may not be a representative sample of the Chinese rural population with stroke. Second, most of the data, such as HRQoL, patterns of sleep, and physical activities, were self-reported and recalled by stroke survivors, and some patients were illiterate; thus, bias might have affected the results. Third, other factors such as the different types of stroke, the severity of the disease, and drug use, which are important factors to consider, were not included, which may have potentially biased our estimates of the impact on HRQoL in patients with stroke. Fourth, we were unable to evaluate within subject changes in HRQoL in patients with stroke and establish causal relations across different factors due to the cross-sectional nature of the study. Fifth, the EQ-5D-5L patient population value set for China was developed from urban areas rather than rural areas, which may introduce bias to our study. Future studies developing EQ-5D-5L patient population value sets for rural areas in China may further enhance the power to determine the level of health state and its influencing factors. Sixth, although a large sample in Henan was recruited in this study, the participants were selected and came from one province, which may limit the generalizability of the findings.

## Conclusion

Patients with stroke in rural areas in China had a low HRQoL. Pain/discomfort was the most frequently reported issue. A lower education level, lower monthly income, lower physical activity intensity, diabetes, anxiety, depression, and poor sleep quality predicted lower health utility index and VAS scores and had effects on each EQ-5D domain score. Rural patients with stroke need to be managed systematically by national public health services.

## Data Availability Statement

The original contributions presented in the study are included in the article/Supplementary Material, further inquiries can be directed to the corresponding author/s.

## Ethics Statement

This study was approved by the Zhengzhou University Life Science Ethics Committee under ethical approval code: [2015] MEC (S128), and written informed consent was obtained from all participants. The patients/participants provided their written informed consent to participate in this study.

## Author Contributions

Y-xM, C-jW, W-hZ, and D-bY contributed to the study conception and design. Material preparation, data collection, and analysis were performed by HW, JH, X-tL, S-xS, and Z-xM. The first draft of the manuscript was written by Y-xM and Z-xZ. All authors commented on previous versions of the manuscript and read and approved the final manuscript.

## Funding

This research was supported by the Foundation of National Key Program of Research and Development of China (Grant No. 2016YFC0900803), China Postdoctoral Science Foundation (Grant No. 2019M652589), National Natural Science Foundation of China (Grant Nos. 72004205, U1304821, 81573243, and 81602925), Science and Technology Innovation Team of University in Henan (22IRTSTHN027) Henan Natural Science Foundation of China (Grant No. 182300410293), Key Research Projects of Higher Education Institutions of Henan (Grant No. 21A320068), Science and Technology Foundation for Innovation Talent of Henan Province (Grant No. 164100510021), Science and Technology Innovation Talents Support Plan of Henan Province Colleges and Universities (Grant Nos. 14HASTIT035 and 17HASTIT048), and High-level Personnel Special Support Project of Zhengzhou University (Grant No. ZDGD13001). The funders had no role in the study design, data collection and analysis, decision to publish, or preparation of the manuscript.

## Conflict of Interest

The authors declare that the research was conducted in the absence of any commercial or financial relationships that could be construed as a potential conflict of interest.

## Publisher's Note

All claims expressed in this article are solely those of the authors and do not necessarily represent those of their affiliated organizations, or those of the publisher, the editors and the reviewers. Any product that may be evaluated in this article, or claim that may be made by its manufacturer, is not guaranteed or endorsed by the publisher.
