# Supplementary material for: Health-Related Quality of Life and Its Related Factors in Survivors of Stroke in Rural China: A Large-Scale Cross-Sectional Study
Source: Front Public Health. 2022 Apr 5;10:810185. doi: 10.3389/fpubh.2022.810185 (PMC9016152; doi:10.3389/fpubh.2022.810185)
Supplement: Supplementary file 1 [file Table_1.DOCX]

Table S1 The characteristics of patients with stroke who were excluded (n=933)

| **Subject characteristics** | n (%) | **Subject characteristics** | n (%) |
| --- | --- | --- | --- |
| **Age(years)** |  | **Physical activity intensity** |  |
| <55 | 141 (15.10) | Mild | 415 (44.50) |
| 55~65 | 334 (35.80) | Moderate | 364 (39.00) |
| 65~ | 458 (49.10) | Intense | 154 (16.50) |
| **Gender** |  | **BMI (n=924)** |  |
| Female | 511(54.80) | <18.5 | 34 (3.60) |
| **Education** |  | 18.5≤BMI<24.0 | 358 (38.40) |
| illiterate | 236 (25.30) | 24.0≤BMI<28.0 | 380 (40.70) |
| Primary school | 319 (34.20) | ≥28.0 | 152 (16.30) |
| Other | 378 (40.50) | **Centripetal obesity (n=929)** |  |
| **Spouse** |  | Yes | 527 (56.50) |
| Yes | 783 (83.90) | **Waist-to-hip ratio (n=929)** |  |
| **Per capita monthly actual income (＄)** |  | abnormal | 659 (70.60) |
| <72 | 420 (45.00) | **Duration of the illness (years)** |  |
| 72~143 | 284 (30.40) | <1 | 143 (15.30) |
| 143~ | 229 (24.50) | ≥1 to < 3 | 268 (28.70) |
| **Smoking status** |  | ≥ 3 to < 5 | 200 (21.40) |
| Never | 648 (69.50) | ≥ 5 | 322 (34.50) |
| Former | 166 (17.80) | **Hypertension** |  |
| Current | 119 (12.80) | Yes | 366 (39.20) |
| **Drink status** |  | **Diabetes mellitus** |  |
| Never | 723 (77.50) | Yes | 119 (12.80) |
| Former | **118 (12.60)** | **Anxiety (n=417)** |  |
| Current | 92 (9.90) | GAD-2≥3 | 46 (11.03) |
| **High-fat diet** |  | **Depression (n=418)** |  |
| Yes | 152 (16.30) | PHQ≥2 | 53 (12.68) |
| **Vegetable and fruit diet** |  | **Sleep quality (n=415)** |  |
| Yes | 357 (38.30) | PSQI＞5 | 181(19.40) |

GAD-2 Generalized Anxiety Disorder Scale-2, PHQ-2 Patient Health Questionnaire-2, PSQI the Pittsburgh Sleep Quality Index
