# Supplementary material for: Health-Related Quality of Life and Its Related Factors in Survivors of Stroke in Rural China: A Large-Scale Cross-Sectional Study
Source: Front Public Health. 2022 Apr 5;10:810185. doi: 10.3389/fpubh.2022.810185 (PMC9016152; doi:10.3389/fpubh.2022.810185)
Supplement: Supplementary file 2 [file Table_2.DOC]

Table S2 Risk factors for having problems in each EQ-5D dimension among survivors with stroke (n=1709): A multiple logistic regression analysis

| Subject characteristics | Mobility  OR (95%CI) | Self-care  OR (95%CI) | Usual activities  OR (95%CI) | Pain/discomfort  OR (95%CI) | Anxiety/depressionOR (95%CI) |
| --- | --- | --- | --- | --- | --- |
| Age(years)(ref.=<55) |  |  |  |  |  |
| 55~65 | 1.28 (0.86-1.90) | 0.89 (0.50-1.58) | 1.04 (0.64-1.69) | 1.17 (0.82-1.68) | 0.82 (0.46-1.45) |
| 65~ | 1.55 (1.05-2.3)* | 1.36 (0.79-2.36) | 1.43 (0.89-2.30) | 1.35 (0.95-1.93) | 1.06 (0.61-1.85) |
| Gender (ref.=Male) |  |  |  |  |  |
| Female | 0.80 (0.56-1.13) | 0.61 (0.38-0.96)* | 0.66 (0.44-0.98)* | 1.3 (0.92-1.84) | 0.84 (0.5-1.39) |
| Education(ref.=Illiterate) |  |  |  |  |  |
| Primary school | 0.82 (0.61-1.10) | 0.75 (0.51-1.11) | 0.87 (0.62-1.21) | 0.95 (0.72-1.25) | 1.04 (0.68-1.58) |
| Junior high school and above | 0.73 (0.53-0.99)* | 0.77 (0.5-1.18) | 0.74 (0.51-1.06) | 0.70 (0.52-0.94)* | 0.94 (0.59-1.5) |
| Per capita monthly actual income (＄)(ref.=<72) | |  |  |  |  |
| 72~143 | 0.86 (0.66-1.11) | 0.86 (0.6-1.23) | 0.91 (0.67-1.23) | 0.81 (0.63-1.05) | 0.89 (0.6-1.32) |
| 143~ | 0.61 (0.45-0.82)* | 0.66 (0.43-1.01) | 0.57 (0.39-0.82)* | 0.81 (0.62-1.07) | 0.66 (0.42-1.04) |
| Smoking status (ref.=Never) |  |  |  |  |  |
| Current | 0.92 (0.6-1.4) | 0.65 (0.36-1.18) | 0.60 (0.36-1.00) | 0.89 (0.59-1.36) | 0.42 (0.20-0.88)* |
| Former | 0.82 (0.54-1.26) | 0.57 (0.31-1.04) | 0.61 (0.37-1.01) | 0.84 (0.55-1.27) | 0.53 (0.27-1.05) |
| Drinking alcohol (ref.=Never) |  |  |  |  |  |
| Current | 1.77 (1.19-2.64)* | 1.63 (0.94-2.81) | 1.81 (1.13-2.89)* | 1.52 (1.02-2.27)* | 0.64 (0.3-1.37) |
| Former | 0.99 (0.65-1.51) | 0.64 (0.32-1.28) | 0.83 (0.48-1.44) | 1.38 (0.92-2.06) | 1.04 (0.51-2.1) |
| High-fat diet (ref.=No) |  |  |  |  |  |
| Yes | 0.81 (0.54-1.22) | 0.65 (0.35-1.21) | 0.68 (0.41-1.14) | 1.12 (0.78-1.61) | 1.22 (0.68-2.22) |
| Vegetable and fruit diet (ref.=No) | |  |  |  |  |
| Yes | 0.79 (0.63-1.00) | 1.09 (0.78-1.5) | 0.89 (0.67-1.17) | 0.84 (0.67-1.05) | 0.53 (0.37-0.77)* |
| Physical activity intensity (ref.=light) | |  |  |  |  |
| Moderate | 0.58 (0.44-0.76)** | 0.47 (0.33-0.69)** | 0.53 (0.38-0.72)** | 0.82 (0.63-1.07) | 0.92 (0.62-1.38) |
| Vigorous | 0.40 (0.30-0.54)** | 0.19 (0.11-0.32)** | 0.29 (0.20-0.43)** | 0.79 (0.6-1.04) | 0.81 (0.52-1.26) |
| BMI (n=1244)(ref.=<18.5) |  |  |  |  |  |
| 18.5≤BMI<24.0 | 0.75 (0.34-1.62) | 1.32 (0.41-4.25) | 1.38 (0.51-3.73) | 1.11 (0.52-2.35) | 0.22 (0.09-0.56)* |
| 24.0≤BMI<28.0 | 0.84 (0.38-1.87) | 1.25 (0.38-4.17) | 1.21 (0.44-3.37) | 1.05 (0.48-2.28) | 0.25 (0.10-0.66)* |
| ≥28.0 | 1.16 (0.51-2.67) | 1.72 (0.5-5.88) | 1.66 (0.58-4.76) | 1.4 (0.63-3.12) | 0.27 (0.10-0.75)* |
| Waist-to-hip ratio (n=1244) (ref.=normal) | |  |  |  |  |
| abnormal | 1.02 (0.75-1.38) | 0.86 (0.56-1.33) | 0.87 (0.60-1.24) | 1.05 (0.79-1.4) | 0.79 (0.49-1.28) |
| Duration of the illness (years) (ref.= <1 ) | |  |  |  |  |
| ≥1 to < 3 | 0.92 (0.62-1.37) | 0.83 (0.48-1.44) | 0.98 (0.60-1.60) | 0.84 (0.59-1.21) | 0.88 (0.5-1.58) |
| ≥ 3 to < 5 | 1.07 (0.7-1.62) | 0.79 (0.43-1.43) | 1.21 (0.73-2.01) | 0.91 (0.62-1.34) | 0.89 (0.48-1.64) |
| ≥ 5 | 1.49 (1.02-2.17)* | 1.26 (0.75-2.11) | 1.60 (1.01-2.52)* | 0.97 (0.68-1.37) | 1.15 (0.66-2) |
| Hypertension (ref.=No) |  |  |  |  |  |
| Yes | 1.29 (1.03-1.63)* | 1.12 (0.82-1.54) | 1.17 (0.89-1.53) | 0.85 (0.68-1.06) | 0.9 (0.63-1.27) |
| Diabetes mellitus (ref.=No) |  |  |  |  |  |
| Yes | 1.45 (1.06-1.98)* | 1.2 (0.79-1.81) | 1.3 (0.91-1.85) | 0.97 (0.71-1.33) | 1.11 (0.69-1.77) |
| Anxiety (n=1246) (ref.=GAD-2<3) | |  |  |  |  |
| GAD-2≥3 | 1.18 (0.71-1.97) | 1.52 (0.80-2.89) | 1.07 (0.61-1.89) | 2.89 (1.77-4.71)** | 3.26 (1.88-5.67)** |
| Depression (n=1246) (ref.=PHQ<2) | |  |  |  |  |
| PHQ≥2 | 2.23 (1.4-3.55)* | 1.74 (0.96-3.13) | 2.75 (1.66-4.55)** | 1.96 (1.25-3.07)* | 4.87 (2.91-8.16)** |
| Sleep quality (n=1229) (ref.=PSQI≤5) | |  |  |  |  |
| PSQI＞5 | 0.58 (0.46-0.75)* | 0.46 (0.34-0.64)** | 0.51 (0.39-0.68)** | 0.50 (0.40-0.63)** | 0.58 (0.41-0.82)* |

GAD-2 Generalized Anxiety Disorder Scale-2, PHQ-2 Patient Health Questionnaire-2, PSQI the Pittsburgh Sleep Quality Index

* *P* <0.05; ** *P* <0.001
