# Supplementary material for: Health-Related Quality of Life and Its Related Factors in Survivors of Stroke in Rural China: A Large-Scale Cross-Sectional Study
Source: Front Public Health. 2022 Apr 5;10:810185. doi: 10.3389/fpubh.2022.810185 (PMC9016152; doi:10.3389/fpubh.2022.810185)
Supplement: Supplementary file 3 [file Table_3.DOC]

Table S3 Tobit regression analyses and Generalized linear model on the health utility index and VAS scores of patients with stroke (n=1709)

| Subject characteristics | Utility index | | | | VAS scores | | | |
| --- | --- | --- | --- | --- | --- | --- | --- | --- |
|  | COE | SE | *t* | *P* | COE | SE | *z* | *P* |
| Age(years) (ref.=<55) |  |  |  |  |  |  |  |  |
| 55~65 | -0.023 | 0.026 | -0.88 | 0.380 | -0.006 | 0.014 | -0.43 | 0.666 |
| 65~ | -0.050 | 0.027 | -1.89 | 0.059 | -0.021 | 0.014 | -1.54 | 0.122 |
| Gender (ref.=Male) |  |  |  |  |  |  |  |  |
| Female | -0.003 | 0.025 | 0.12 | 0.903 | 0.016 | 0.138 | 1.15 | 0.249 |
| Education (ref.=Illiterate) |  |  |  |  |  |  |  |  |
| Primary school | 0.019 | 0.021 | 0.89 | 0.372 | 0.012 | 0.011 | 1.03 | 0.305 |
| Junior high school and above | 0.045 | 0.022 | 2.05 | **0.041*** | 0.024 | 0.012 | 1.97 | **0.049*** |
| Per capita monthly actual income (＄) (ref.=<72) | |  |  |  |  |  |  |  |
| 72~143 | 0.312 | 0.018 | 1.66 | 0.097 | 0.023 | 0.010 | 2.22 | **0.027*** |
| 143~ | 0.071 | 0.021 | 3.14 | **0.001*** | 0.035 | 0.011 | 3.17 | **0.002*** |
| Smoking (ref.=Never) |  |  |  |  |  |  |  |  |
| Current | 0.039 | 0.031 | 1.27 | 0.206 | 0.026 | 0.017 | 1.56 | 0.119 |
| Former | 0.059 | 0.031 | 1.92 | 0.055 | 0.032 | 0.016 | 1.99 | **0.046*** |
| Drinking alcohol(ref.=Never) |  |  |  |  |  |  |  |  |
| Current | -0.064 | 0.029 | -2.19 | **0.028*** | -0.019 | 0.016 | -1.21 | 0.226 |
| Former | -0.037 | 0.030 | -1.25 | 0.213 | -0.009 | 0.016 | -0.58 | 0.562 |
| High-fat diet(ref.=No) |  |  |  |  |  |  |  |  |
| Yes | 0.011 | 0.028 | 0.38 | 0.703 | 0.010 | 0.015 | 0.70 | 0.485 |
| Vegetable and fruit diet (ref.=No) |  |  |  |  |  |  |  |  |
| Yes | 0.023 | 0.017 | 1.39 | 0.166 | 0.011 | 0.009 | 1.23 | 0.218 |
| Physical activity intensity (ref.=light) |  |  |  |  |  |  |  |  |
| Moderate | 0.085 | 0.019 | 4.41 | **<0.001**** | 0.057 | 0.011 | 5.41 | **<0.001**** |
| Vigorous | 0.145 | 0.021 | 6.97 | **<0.001**** | 0.082 | 0.011 | 7.49 | **<0.001**** |
| BMI (n=1244)(ref.=<18.5) |  |  |  |  |  |  |  |  |
| 18.5≤BMI<24.0 | 0.057 | 0.055 | 1.04 | 0.297 | 0.034 | 0.031 | 1.12 | 0.263 |
| 24.0≤BMI<28.0 | 0.047 | 0.057 | 0.83 | 0.406 | 0.026 | 0.032 | 0.80 | 0.425 |
| ≥28.0 | -0.001 | 0.059 | -0.02 | 0.988 | -0.001 | 0.033 | -0.02 | 0.986 |
| Waist-to-hip ratio (n=1244) (ref.=Normal) | |  |  |  |  |  |  |  |
| Abnormal | 0.007 | 0.021 | 0.31 | 0.760 | 0.002 | 0.012 | 0.20 | 0.838 |
| Duration of the illness (years) (ref.= <1 ) |  |  |  |  |  |  |  |  |
| ≥1 to < 3 | 0.014 | 0.028 | 0.53 | 0.596 | 0.007 | 0.015 | 0.45 | 0.650 |
| ≥ 3 to < 5 | -0.006 | 0.029 | -0.19 | 0.846 | -0.002 | 0.016 | -0.13 | 0.896 |
| ≥ 5 | -0.049 | 0.027 | -1.84 | 0.066 | -0.026 | 0.014 | -1.86 | 0.063 |
| Hypertension (ref.=No) |  |  |  |  |  |  |  |  |
| Yes | -0.005 | 0.016 | -0.33 | 0.741 | -0.002 | 0.009 | -0.28 | 0.778 |
| Diabetes mellitus (ref.=No) |  |  |  |  |  |  |  |  |
| Yes | -0.061 | 0.023 | -2.68 | **0.008*** | -0.036 | 0.013 | -2.83 | **0.005*** |
| Anxiety (ref.=GAD-2<3) |  |  |  |  |  |  |  |  |
| GAD-2≥3 | -0.138 | 0.035 | -3.96 | **<0.001**** | -0.076 | 0.021 | -3.71 | **<0.001**** |
| Depression (ref.=PHQ<2) |  |  |  |  |  |  |  |  |
| PHQ≥2 | -0.191 | 0.033 | -5.85 | **<0.001**** | -0.133 | 0.019 | -6.93 | **<0.001**** |
| Sleep quality (n=1693) (ref.=PSQI≤5) |  |  |  |  |  |  |  |  |
| PSQI＞5 | -0.111 | 0.018 | 6.31 | **<0.001**** | -0.059 | 0.010 | 5.98 | **<0.001**** |

VAS: Visual Analogue Scale

GAD-2 Generalized Anxiety Disorder Scale-2, PHQ-2 Patient Health Questionnaire-2, PSQI the Pittsburgh Sleep Quality Index

* *P* <0.05; ** *P* <0.001
